# Aesthetic and Functional Rehabilitation of Patients with Genetic Microdontia: A Multidisciplinary Approach

**DOI:** 10.3390/healthcare10030485

**Published:** 2022-03-05

**Authors:** Cindy Batisse, Pierre-Yves Cousson, Emmanuel Nicolas, Marion Bessadet

**Affiliations:** 1 Centre de Recherche en Odontologie Clinique (CROC), Université Clermont Auvergne, F-63000 Clermont-Ferrand, France; p-yves.cousson@uca.fr (P.-Y.C.); emmanuel.nicolas@uca.fr (E.N.); marion.bessadet@uca.fr (M.B.); 2 Service d’Odontologie, CHU Clermont-Ferrand, F-63003 Clermont-Ferrand, France

**Keywords:** microdontia, CAD/CAM, general anesthesia, special needs

## Abstract

This case report presents the treatment of a 33-year-old patient with a genetic, generalized microdontia of permanent teeth. Microdontia is a developmental dental anomaly of the teeth characterized by a decrease in their size. In the literature, treatment has been multidisciplinary, often utilizing orthodontics and implantology. However, for adult patients with generalized microdontia who do not benefit from these treatments as much, a combination of adhesive dentistry, endodontics and removable prostheses remains a therapeutic alternative to consider. Given the specificities of the patient and the nature of the care, the objective of this treatment option was to manage the patient’s comfort while guaranteeing quality of care. A feature of this treatment was the use of general anesthesia for endodontic treatments and exodontia. Chairside CAD/CAM and adhesive dentistry reduced the chairside time and preserved healthy dental tissue.

## 1. Introduction

Microdontia is a type of developmental dental anomaly in which teeth appear smaller than usual. It can affect one or more teeth. Shafer et al. described three types of microdontia: single tooth microdontia (usually the maxillary lateral incisor or third molars); relative generalized microdontia due to relatively large jaws; and true generalized microdontia involving all teeth [1]. Microdontia may affect the entire tooth, or only part of the tooth, i.e., the crown or the root according to the Classification of Bargale, et al. [2]. The prevalence of microdontia ranges from 1.5 to 2%. It affects both deciduous and permanent teeth with a prevalence of 0.5% and 2.5% respectively [3]. This anomaly has important aesthetic (presence of enlarged diastemas) and functional (malocclusion, food retention) repercussions.

Microdontia’s etiology remains poorly understood. It may be of genetic and/or environmental origin [4].

The treatment of localized microdontia generally consists of orthodontic treatment to idealize the dental positions and then direct or indirect restoration of the teeth. Exodontia is also an alternative, restoring function and aesthetics utilizing implant, bonded, and/or traditional fixed partial dental prostheses. The use of removable partial dentures (RPD) is rarely possible because the partial edentulism is often minor. The management of microdontia is therefore often multidisciplinary and the choice of therapy must consider the extent and severity of the microdontia, the patient’s age and further growth potential, compliance, and aesthetic and functional expectations [5]. The treatment of generalized microdontia in adults is poorly documented. The objective of this case report is to present an example of a therapeutic approach to a generalized microdontia of permanent teeth in a healthy adult.

## 2. Case Presentation

### 2.1. Clinical and Radiological Examination

The patient is a 33-year-old man with generalized microdontia of genetic origin (autosomal dominant transmission). His microdontia is not associated with a syndrome, but rather idiopathic. He had no other associated pathology. He consulted the Odontology Department of the Clermont Ferrand University Hospital (France) for aesthetic and functional oral rehabilitation. He was anxious about undergoing dental therapy, but wanted a more aesthetic and functional oral cavity. His oral quality of life was evaluated using the GOHAI (General Oral Health Assessment Index) questionnaire [6]. He obtained a score of 33/60 which corresponds to a poor oral quality of life. His financial resources were low.

**Figure 1:**
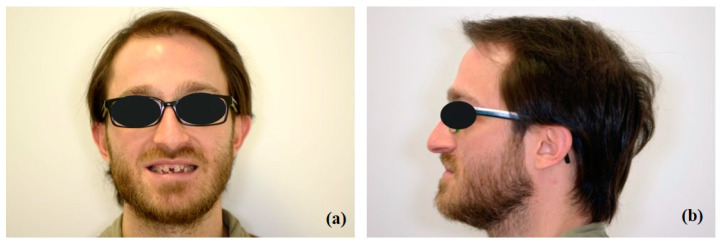
Extraoral examination: (**a**) front view; (**b**) side view.

The extraoral examination revealed: from a frontal view, an unaesthetic smile related to the presence of diastemas (Figure 1a); from a profile view, a lack of support of the lower lip associated with a hypo development of the maxilla and partial edentulism in the mandibular anterior region (Figure 1b).

**Figure 2:**
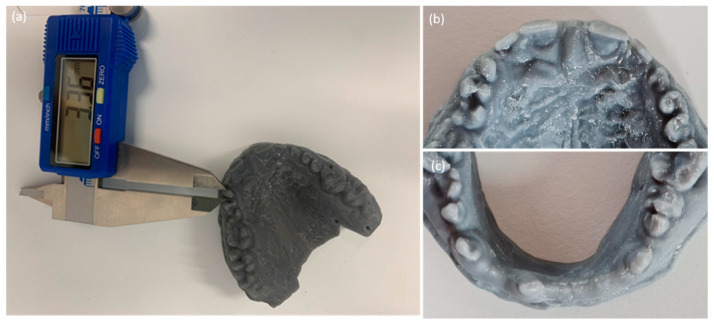
Resin printed study models illustrating microdontia and the abnormal morphology of the clinical crowns: (**a**) maxillary model; (**b**) model of anterior maxillary segment; (**c**) model of anterior mandibular segment.

Intraoral examination revealed generalized microdontia with localized hypodontia, severely affecting the anterior segments. The premolars exhibited unusually shaped crowns as well as small roots. The molars were less affected. The incisal edges of the maxillary incisors and the canine cusp tips were retroclined palatally or lingually. The canines had a very tapered appearance. The mandibular premolars had a characteristic conical shape with a pronounced buccal cusp and a diminished lingual cusp (Figure 2).

**Figure 3:**
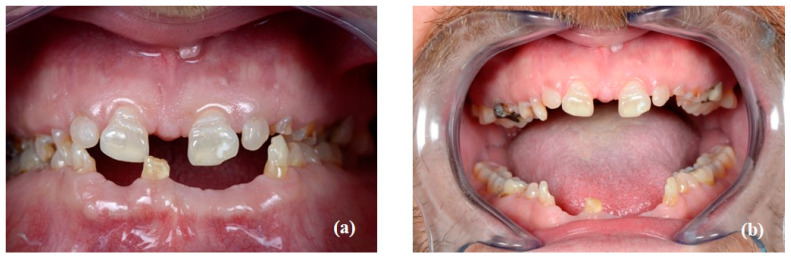
Intraoral examination: frontal view (**a**) dental arches in occlusion; (**b**) arches in full opening.

The patient’s oral hygiene showed a moderate amount of plaque (Silness–Loe plaque index: 2, [7]) in the presence a healthy periodontium. Twenty-nine teeth were present (32, 31, and 42 were missing due to spontaneous exfoliations). Teeth numbers 28 and 46 presented with carious lesions and many restorations were already present on the molars (16, 26, 36, 37, 47, and 48), indicating a high caries risk (Figure 3).

**Figure 4:**
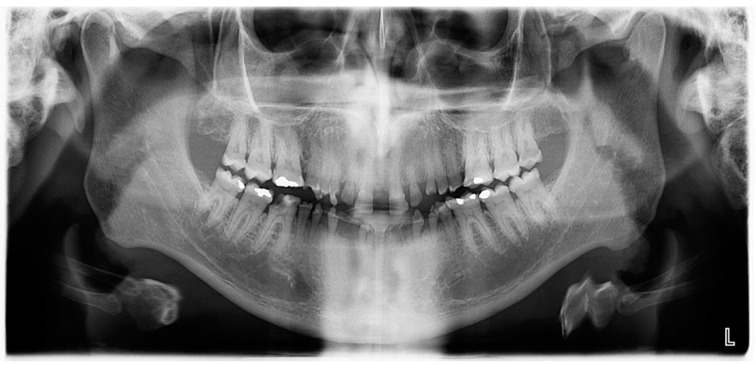
Panoramic radiograph before treatment.

**Figure 5:**
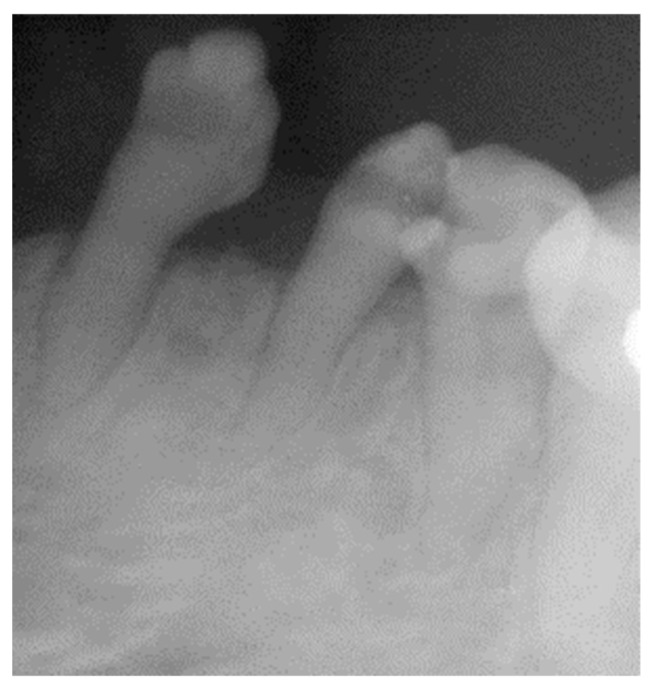
Radiographs of 33, 34 and 35 showing the small size of the roots of the canines and premolars and the abnormal dental morphology of their clinical crowns.

Radiographic examination revealed that the roots of the anterior teeth were partic-ularly short (Figure 4 and Figure 5). The diagnosis of 46 was acute apical abscess. The prognosis was unfavorable confirmed by radiography showing a severe coronal decay.

A study of the diagnostic casts on the articulator revealed a lack of space between and around the residual ridges (Figure 3). In maximal intercuspal position, the maxillary incisors were very close to the soft tissue of the opposing arch. A diagnostic tooth arrangement incorporating a 2–3 mm increase in occlusal vertical dimension (OVD) was performed. This increase in OVD space allowed for more favorable smile design and restorative materials.

### 2.2. Therapeutic Objectives and Proposals

In order to satisfy the patient’s aesthetic needs, the severity and location of the diastemas needed to be considered. Evaluation of the size of the roots of the maxillary and mandibular incisors, canines and premolars, showed that their restoration by fixed prosthesis could not be considered. To replace these teeth (15, 14, 13, 12, 11, 21, 22, 23, 24, and 25 in the maxilla and 35, 34, 33, 32, 31, 41, 42, 43, 45, and 46 in the mandible), transitional removable partial dentures (TRDP), followed by definitive removable partial dental prostheses (RPD) were proposed and accepted by the patient. An implant treatment was also proposed to the patient but was not accepted due to financial reasons. As the diagnostic tooth arrangement showed, a slight increase in vertical dimension was necessary to allow for the placement of anterior denture teeth on the removable prostheses. To maintain posterior molars in occlusion, hybrid resin/ceramic onlays bonded to the molars (16, 17, 18, 26, 27, 28, 36, 37, 38, 47, 48) were also proposed to the patient. In the anterior segments, teeth with excessively short roots were extracted. The other teeth were devitalized endodontically, the clinical crown was amputated, and the endo access was restored with bonded amalgam. This method allowed more room for restorative materials, as the endodontically treated teeth were utilized as overdenture abutments under the removable dentures. Retaining the roots allowed tooth-born support, rather than tissue born support, of the prosthesis, as well as preservation the alveolar bone if an implant prosthetic rehabilitation is performed in the future.

### 2.3. Treatment

**Figure 6:**
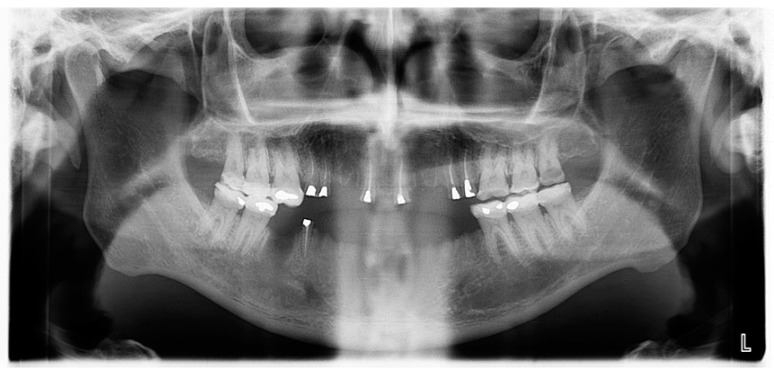
Panoramic radiograph after procedures performed under general anesthesia.

**Figure 7:**
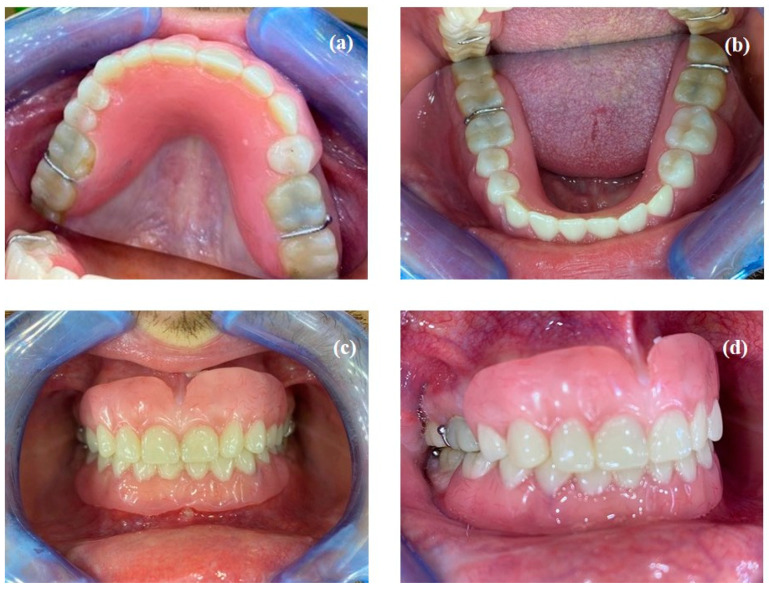
Resin mock-up and transitional removable partial dentures, (**a**) maxillary view; (**b**) mandibular view; (**c**,**d**) arches in occlusion.

**Figure 8:**
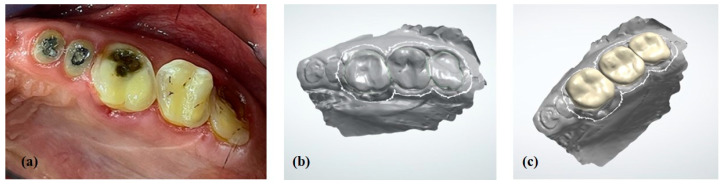
Onlays in maxillary posterior segment 1: (**a**) Occlusal preparations; (**b**) digital cast; (**c**) design of onlays.

**Figure 9:**
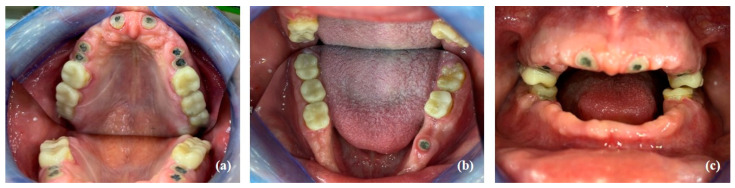
(**a**) maxillary onlays; (**b**) mandibular onlays; (**c**) posterior onlays in occlusion.

**Figure 10:**
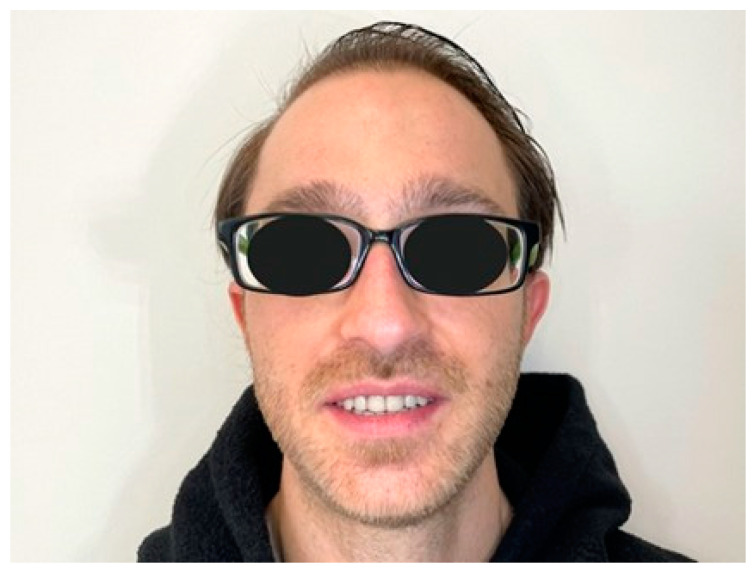
Patient smile after treatment.

The treatment plan was performed in four steps:

**(1)**  Validation of the setup in the mouth and fabrication of the transitional removable partial dentures: the increase in OVD was accomplished by transferring a composite resin mock-up (Voco StructurPremium, Cuxhaven, Germany) to the molars in the mouth. The aesthetic evaluation of the increased OVD was verified on the day of placement (consistency in profile). However, the functional verification was done after one week of wearing the mock-up considering the patient’s feedback, phonetics and the absence of pain, clicking or cracking in the temporomandibular joints. Two alginate impressions were made using custom trays in the maxilla and mandible with and without mock-ups in place, and silicone maxillomandibular records (VPS Hydro Bite HENRY SCHEIN, Melville, NY, USA) were made in order to finalize the transitional removable partial dentures.
**(2)**  Pre-prosthetic care: exodontia, endodontic and restorative treatments were performed under general anesthesia. The following procedures were performed: endodontic treatment and bonded amalgam restorations for overdenture abutments of teeth numbers 11, 14, 15, 21, 24, 25 and 45 and exodontia of 46, 44, 43, 41, 33, 34, 35, 23, 22, 13 and 12 (Figure 6). The TRPD were placed on the day of surgery and a postoperative consultation was performed the following day to evaluate and adjust the prostheses (Figure 7).
**(3)**  Fabrication of fixed prostheses: the onlays were fabricated in two clinical sessions using chairside CAD/CAM techniques with a PrimeScan™ intraoral scanner (Dentsply Sirona, York, PA, USA). Two opposing quadrants were restored each session (left posterior segments 2 and 3, then right posterior segments 1 and 4).
  Preliminary digital impressions with the mock-up in place were made to ensure that the coronal anatomy of the future onlays were identical to the mock-up. The preparation for the onlays consisted of the removal of the mock-up and the occlusal restorations, the curettage of any decay and the preparation of any existing undercuts. Finally, an occlusal reduction was performed to provide sufficient height for the restorative material of the onlays (Figure 8). The onlays were then digitally designed in the same clinical session (Figure 8).
  The choice of milling material was justified by the very minimal restoration thickness (due to the small occlusal vertical dimension increase). All onlays were therefore milled from hybrid resin/ceramic blocks (VITA Enamic^®,^ Bad Säckingen, Germany). The onlays were bonded intraorally using rubber dam isolation and a light-curing adhesive with no adhesion potential (Variolink^®^ Esthetic, Ivoclar, Saint jorioz, France) in accordance with the manufacturer’s instructions for use (Figure 9).
**(4)**  Fabrication of definitive removable partial dentures: the definitive prostheses were fabricated using the conventional technique from functional impressions. The patient’s new smile is shown in Figure 10.

Two weeks after the end of treatment, an increase in the GOHAI score was noted, as compared to the initial situation, with a score of 44/60. Three months after the end of the treatment the final score was 53/60, which corresponds to an oral quality of life termed as adequate according to the GOHAI scale.

## 3. Discussion

Some of the patient’s clinical crowns were amputated due to the inadequate the size of their roots, i.e., those teeth with a poor support prognosis in the long term. While their existing crown-to-root ratio was acceptable because both the roots and crowns were undersized, the coronal volume and height required to restore them would have been disproportionate with respect to the root length. The restorations would have been over-contoured with a risk of permanent gingival inflammation. Some of the patient’s teeth were extracted due to the extremely short length of their roots or unrestorable caries.

Orthodontic treatment is frequently described in the management of microdontia [2,8]. In this case, orthodontic treatment of the two maxillary central incisors combined with an implant-supported rehabilitation could have been considered. This therapeutic solution was not chosen by the patient due to financial reasons. However, in the event the patient wishes future implant treatment, the roots of 11, 14, 15, 21, 24, 25 and 45 were retained as overdenture abutments under the prosthesis to preserve the alveolar bone [9]. Preservation of endodontically treated roots under a prosthesis allows to preserve some proprioception through periodontal ligament nerve receptors. It is a source of sensory input that initiates a jaw opening reflex [10] and improves chewing efficiency compared to edentulous patients rehabilitated with complete dentures or implant-supported dentures [11]. Another advantage of retaining non-vital teeth as overdenture abutments is the limitation of bone resorption [12]. A recent retrospective clinical study showed that root-retained overdentures are “a viable treatment option in partially dentate subjects, even over long-term periods” despite biological complications (root caries, apical lesions, fractured roots) [13].

General anesthesia (GA) was chosen in order to shorten the duration of treatment for an anxious patient. A significant number of lengthy treatments were necessary (endodontic therapy and exodontia) and the microdontia made them technically more difficult. Also, the partial removable transitional prostheses could be inserted at the end of the medical intervention without the patient being exhausted. General anesthesia made the treatment much more comfortable and easier for both the anxious patient and the practitioner. Long repeated sessions could have led to a loss of motivation for the patient [14].

The use of direct CAD/CAM saved time. In addition, bonding the onlays on the same day as tooth preparation allowed for immediate dentin sealing (IDS), which offers many biological advantages, such as waterproofing of the exposed dentin surface and eliminating postoperative sensitivity. IDS also increases the bonding values of the restorative material to tooth structure [15].

The choice of material for the onlays was a hybrid resin/ceramic. Its mechanical properties are closer to natural tooth structure compared to ceramics. This material is more resistant to wear than composites and its resin matrix makes the material less brittle than monolithic ceramics [16]. In this clinical case, the available height for the posterior restoration available was minimal, so the mechanical properties of Enamic^®^ made it the material of choice. This material allowed the machining of thin prosthetic restorations without threat of breakage. The use of adhesive dentistry in this clinical case made it possible to dispense with retentive peripheral preparations and thus preserve valuable tooth structure. Retention was ensured by chemical and micromechanical adhesion and thus, a significant amount of tooth structure could be preserved thanks to a minimal tooth preparation technique.

In this clinical situation, the treatment resulted in a significant improvement in the patient’s oral quality of life. An improvement in the patient’s aesthetic appearance was noted directly after the placement of the prostheses. In terms of mastication, no improvement was noted during the first weeks of wearing the prostheses, certainly due to the lack of patient adaptation to removable prostheses. Indeed, the patient’s adjustment to wearing RPDs was not immediate and is evidenced by the clear increase in the GOHAI score after a few weeks of wear [17].

The prevalence of generalized microdontia in adults is very low and therefore its management is very poorly documented. There are no published reviews or studies on the therapeutic approach of microdontia in adults, let alone large-scale, epidemiological studies of high evidentiary significance. However, this clinical management philosophy and treatment, despite its low level of evidence, can serve as an example of the multidisciplinary therapeutic approach that can be applied to patients with generalized microdontia of the permanent dentition.

## 4. Conclusions

This clinical case presentation is an example of functional and aesthetic rehabilitation in a patient with generalized microdontia. The use of general anesthesia and CAD/CAM facilitated implementation of the treatment plan and reduced the length of the treatment time. Adhesive dentistry has made it possible to place prosthetic restorations, using minimal preparations which preserved the precious small amount of existing tooth structure.
